# A novel anti-mouse CCR7 monoclonal antibody, C_7_Mab-7, demonstrates high sensitivity in flow cytometry, western blot, and immunohistochemistry

**DOI:** 10.1016/j.bbrep.2025.101948

**Published:** 2025-02-11

**Authors:** Hiroyuki Satofuka, Hiroyuki Suzuki, Tomohiro Tanaka, Rena Ubukata, Miu Hirose, Haruto Yamamoto, Yu Kaneko, Shiori Fujisawa, Guanjie Li, Mika K. Kaneko, Yukinari Kato

**Affiliations:** Department of Antibody Drug Development, Tohoku University Graduate School of Medicine, 2-1 Seiryo-machi, Aoba-ku, Sendai, Miyagi, 980-8575, Japan

**Keywords:** Mouse CCR7, Monoclonal antibody, Cell-based immunization and screening, Flow cytometry, Western blot, Immunohistochemistry

## Abstract

C–C chemokine receptor type 7 (CCR7) is a member of the G protein-coupled receptor family and functions as a lymph node-homing receptor for immune cells. Upon ligand binding, CCR7 promotes the migration of immune cells to secondary lymphoid organs. In cancers, CCR7 has been revealed as a critical molecule in lymph node metastasis. Consequently, anti-CCR7 monoclonal antibodies (mAbs) have been developed as cancer therapeutic agents. In this study, we established an anti-mouse CCR7 (mCCR7) mAb, C_7_Mab-7 (rat IgG_1_, kappa) using the Cell-Based Immunization and Screening (CBIS) method. C_7_Mab-7 demonstrated high sensitivity in flow cytometry. The dissociation constant (*K*_D_) value of C_7_Mab-7 was determined to be 2.5 × 10⁻⁹ M for mCCR7-overexpressed Chinese hamster ovary-K1 (CHO/mCCR7) cells. Furthermore, C_7_Mab-7 detected mCCR7 with high sensitivity in western blot and immunohistochemistry. C_7_Mab-7, developed by the CBIS method, accelerates the development of CCR7-targeted antibody therapies and cancer diagnostics.

## Highlights

• CCR7 is a promising target of antibody therapy for cancers.
• A novel anti-mouse CCR7 monoclonal antibody, C_7_Mab-7, showed high affinity in flow cytometry.
• C_7_Mab-7 is also available for western blot and immunohistochemistry.
• C_7_Mab-7 is expected to detect mouse CCR7-positive cells and may be useful to obtain the proof of concept in preclinical studies.

## 1 Introduction

C–C chemokine receptor type 7 (CCR7), also called CD197, is a receptor that facilitates the homing of immune cells, such as naïve T cells [1], dendritic cells [2,3], B cells [4,5], natural killer cells [6], and memory T cells [7] to lymph nodes [8,9]. The interactions between CCR7 and its ligands, CC-chemokine ligand (CCL) 19 and CCL21, promote the migration of CCR7-expressing cells to secondary lymphoid organs, such as the lymph nodes, thymus, and spleen [[8], [9], [10], [11], [12]]. CCL19 and CCL21 are constitutively expressed on the high endothelial venules of lymph nodes [13]. These chemokines recruit CCR7-expressing cells into the lymph nodes to maintain the immune system. Additionally, the transcription of CCR7 is upregulated by transcription factors, such as NF-κB and AP-1, which are activated by inflammatory cytokines produced in inflammatory sites and tumor microenvironment [14,15].

Lymph node metastasis is an important parameter to determine the prognosis of cancer patients [11,16]. The CCR7-CCL19 and CCR7-CCL21 axes have been shown to promote lymph node metastasis in CCR7-expressing breast cancer cells [17,18]. Furthermore, the elevated expression of CCR7 correlates with lymph node metastasis in various solid cancers, such as colorectal [19], esophageal [20], gastric [21], pancreatic [22], oral [23], thyroid [24], and non-melanoma skin cancers [25]. In addition, CCR7 is recognized as a therapeutic target in hematologic malignancies, such as T-cell prolymphocytic leukemia [26], B-cell chronic lymphocytic leukemia [27], and non-Hodgkin's lymphoma [28].

Blocking (non-activating) monoclonal antibodies (mAbs) that target CCR7 or its ligands have demonstrated high antitumor efficacy in preclinical models of hematologic malignancies, such as B-cell acute lymphoblastic leukemia [29], chronic lymphocytic leukemia [[30], [31], [32]], mantle cell lymphoma [33], T-cell acute lymphoblastic leukemia [29,34], and T-cell prolymphocytic leukemia [26]. In addition, targeting CCR7 with an antibody-drug conjugate has been reported as a promising therapeutic strategy for lymphoid malignancies [35].

CCL21 and CCL19 expressed in several stromal cells [36] may exhibit antitumor activities at primary tumor sites because these chemokines induce the recruitment of CCR7-expressing activated dendritic cells to the tumor site [2,3,5,37]. In this context, anti-mouse CCR7 (mCCR7) mAbs that specifically bind to the endogenous receptor are needed to evaluate efficacy and safety in animal models, thereby accelerating the development of CCR7-targeting therapeutics.

We have developed various mAbs against membrane proteins using the Cell-Based Immunization and Screening (CBIS) method [[38], [39], [40], [41], [42], [43], [44], [45]]. This method effectively produces mAbs that recognize conformational structures. The obtained mAbs are suitable for flow cytometry because the target molecule is expressed as an antigen on the surface of immunized cells. Furthermore, some of the obtained mAbs are also suitable for western blot and immunohistochemistry. This allows simultaneous contributions to the development of therapeutic and diagnostic applications.

Among the anti-mCCR7 mAbs developed to date, 4B12 (rat IgG_2a_, kappa) is frequently utilized to detect the intact structure of mCCR7 [2], while E75 (rabbit IgG) is used for western blot [46] and EPR23192-57 (rabbit IgG) is used for immunohistochemistry [47]. In addition, anti-human CCR7 mAbs, such as 3D12 (rat IgG_2a_, kappa [48]) and ARC0231 (rabbit IgG), have been reported to cross-react with mCCR7. However, no anti-mCCR7 mAbs are currently suitable for use in all three applications, such as flow cytometry, western blot, and immunohistochemistry. In this study, we employed the CBIS method to generate a highly versatile anti-mCCR7 mAb.

## 2 Materials and methods

### 2.1 Cell lines

Chinese hamster ovary (CHO)–K1, mouse myeloma P3X63Ag8.U1 (P3U1), and human glioblastoma LN229 cells were obtained from the American Type Culture Collection (ATCC, Manassas, VA, USA). LN229 cells were maintained in Dulbecco's Modified Eagle Medium (DMEM) supplemented with 100 U/mL penicillin, 100 μg/mL streptomycin, 0.25 μg/mL amphotericin B (Nacalai Tesque, Inc., Kyoto, Japan), and 10 % heat-inactivated fetal bovine serum (FBS; Thermo Fisher Scientific, Inc., Waltham, MA, USA). CHO–K1 and P3U1 cells were maintained in Roswell Park Memorial Institute (RPMI)-1640 medium (Nacalai Tesque, Inc.) with the same antibiotics described above and 10% heat-inactivated FBS. All cells were cultured in a humidified incubator at 37°C with 5% CO_2_.

### 2.2 Plasmid construction and establishment of stable transfectants

The synthesized DNA (Eurofins Genomics KK, Tokyo, Japan) encoding mouse CCR7 (Accession No.: NM_007719) was subcloned into pCAG-Ble-PAcH vector (FUJIFILM Wako Pure Chemical Corporation, Osaka, Japan) using the In-Fusion HD Cloning Kit (Takara Bio, Inc., Shiga, Japan). The constructed vector was designated pCAG-mCCR7-PA. Using the Neon transfection system, the plasmid was transfected into CHO–K1 and LN229 cells (Thermo Fisher Scientific, Inc.). Transfectants expressing the target gene were detected using the anti-mCCR7 mAb 4B12 (rat IgG_2a_, kappa, BioLegend, San Diego, CA, USA). Stable transfectants were isolated by cell sorting (SH800 Cell Sorter, Sony Corporation, Tokyo, Japan), and cell lines were established by introducing pCAG-mCCR7-PA into CHO–K1 cells (CHO/mCCR7 cells) and LN229 cells (LN229/mCCR7 cells). These cell lines were maintained in medium containing 0.5 mg/mL Zeocin (InvivoGen, San Diego, CA, USA).

### 2.3 Hybridoma production

Hybridoma production was performed as previously described [49]. A 5-week-old female Sprague-Dawley rat (Jcl: SD rat, CLEA Japan, Tokyo, Japan) was housed under specific pathogen-free conditions. All animal experiments were approved by the Animal Care and Use Committee of Tohoku University (Permit number: 2022MdA-001) and followed the relevant guidelines to minimize animal suffering and distress in the laboratory. The rat was immunized intraperitoneally with LN229/mCCR7 cells (1 × 10⁹ cells/injection) with Alhydrogel adjuvant 2 % (InvivoGen). Following three weekly immunizations, a booster injection was administered two days prior to the harvesting of spleen cells. Hybridomas were generated by fusing spleen cells with P3U1 cells using polyethylene glycol 1500 (Roche Diagnostics, Indianapolis, IN, USA). RPMI-1640 medium supplemented with hypoxanthine, aminopterin, and thymidine (HAT; Thermo Fisher Scientific, Inc.) was used to select hybridomas. Supernatants that were negative for CHO–K1 cells but positive for CHO/mCCR7 cells were identified using flow cytometry (SA3800 Cell Analyzer, Sony Corporation). To produce purified mAbs, hybridomas were cultured in Hybridoma-SFM (Thermo Fisher Scientific, Inc.), and the mAbs were purified using Ab-Capcher (ProteNova Inc., Kagawa, Japan).

### 2.4 Flow cytometry

Cells were detached using 1 mM ethylenediaminetetraacetic acid (EDTA; Nacalai Tesque, Inc.) to prevent enzymatic degradation of surface proteins. The cells were washed with 0.1% bovine serum albumin (BSA) in phosphate-buffered saline (PBS) (blocking buffer) and incubated with mAbs at 4°C for 30 min. After washing, the cells were incubated with anti-rat IgG (H+L)-Alexa Fluor 488 conjugate (1:2,000 dilution; Cell Signaling Technology, Inc., Danvers, MA, USA) at 4°C for 30 min. Data were collected using the SA3800 Cell Analyzer and analyzed using FlowJo software (BD Biosciences, Franklin Lakes, NJ, USA).

### 2.5 Determination of dissociation constant value using flow cytometry

CHO/mCCR7 cells were treated with serial dilutions of C_7_Mab-7 and 4B12 (0.005 to 10 μg/mL). The cells were stained with anti-rat IgG (H+L)-Alexa Fluor 488 conjugate (1:200 dilution) at 4°C for 30 min. Data were collected using the SA3800 Cell Analyzer and analyzed using FlowJo software. The geometric mean fluorescence intensity of CHO/mCCR7 at each concentration of mAbs was plotted. By fitting one-site binding models in GraphPad Prism 6 software (GraphPad Software, Inc., La Jolla, CA, USA), the *K*_D_ values of C_7_Mab-7 and 4B12 for CHO/mCCR7 were determined.

### 2.6 Western blot analysis

Whole-cell lysates (10 μg of protein per lane) were separated using 5–20% polyacrylamide gels (FUJIFILM Wako Pure Chemical Corporation). The separated proteins were transferred onto polyvinylidene difluoride (PVDF) membranes (Merck KGaA, Darmstadt, Germany). The membranes were blocked with 4% skim milk (Nacalai Tesque, Inc.) in PBST. Subsequently, the membranes were incubated with 1 μg/mL of C_7_Mab-7, 4B12, or an anti-β-actin mAb (AC-15; Sigma-Aldrich Corporation), followed by incubation with rabbit anti-rat IgG conjugated with horseradish peroxidase (1:20,000 dilution; Merck KGaA) or rabbit anti-mouse immunoglobulins conjugated with horseradish peroxidase (1:2,000 dilution; Agilent Technologies, Inc., Santa Clara, CA, USA). Secondary antibodies were matched to the host species of each primary antibody. Chemiluminescence signals were developed using the ImmunoStar LD (FUJIFILM Wako Pure Chemical Corporation) or Pierce ECL Plus Western Blotting Substrate (Thermo Fisher Scientific, Inc.) and detected with a Sayaca-Imager (DRC Co., Ltd., Tokyo, Japan).

### 2.7 Immunohistochemical analysis

Cell blocks were prepared using iPGell (Genostaff Co., Ltd., Tokyo, Japan) and fixed in a 4% paraformaldehyde phosphate buffer solution (FUJIFILM Wako Pure Chemical Corporation). The blocks were processed to create 4-μm-thick paraffin-embedded cell sections. The sections were autoclaved in citrate buffer (pH 6.0; Nichirei Biosciences, Inc., Tokyo, Japan) for 20 min. These sections were blocked with SuperBlock T20 Blocking Buffer (Thermo Fisher Scientific Inc.), incubated with C_7_Mab-7 (1 μg/mL) at room temperature for 1 h, and subsequently treated with Histofine Simple Stain Mouse MAX PO (Rat) (Nichirei Biosciences, Inc.) for 30 min at room temperature. Color development was achieved using 3,3'-diaminobenzidine tetrahydrochloride (DAB; Agilent Technologies Inc.), and counterstained with hematoxylin (Merck KGaA).

## 3 Results

### 3.1 Development of anti-mouse CCR7 mAbs

**Fig. 1:**
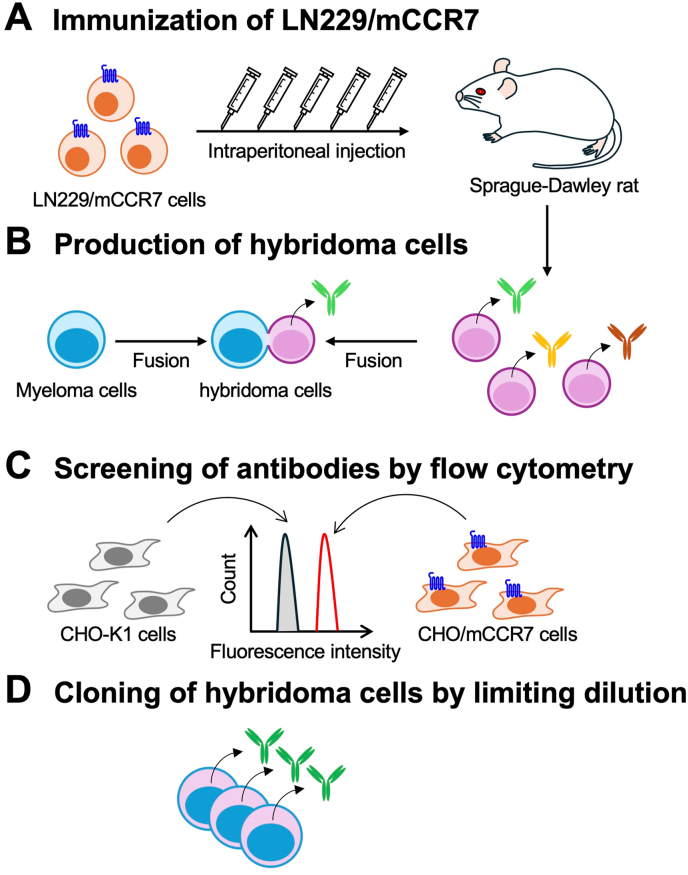
Schematic representation of anti-mCCR7 mAbs production. (A) LN229/mCCR7 cells were intraperitoneally injected into a Sprague-Dawley rat. (B) Following immunization, spleen cells were harvested and fused with P3U1 cells. (C) Hybridoma supernatants were screened for anti-mCCR7-specific mAbs using flow cytometry with CHO/mCCR7 and parental CHO–K1 cells. (D) Antigen-specific mAb-producing hybridomas were isolated by the limiting dilution method.

A Jcl: SD rat was immunized with LN229/mCCR7 cells (Fig. 1A). Spleen cells were harvested from the immunized rat, and hybridomas were produced by fusion with P3U1 cells (Fig. 1B). These hybridomas were seeded into 96-well plates. After colony formation, supernatants were collected and analyzed using a flow cytometry-based high-throughput screening to identify supernatants that were positive for CHO/mCCR7 cells but negative for CHO–K1 cells (Fig. 1C). Anti-mCCR7 mAb-producing hybridomas were subsequently cloned by limiting dilution, and C_7_Mab-7 (rat IgG_1_, kappa) was finally established (Fig. 1D).

### 3.2 Flow cytometry using C_7_Mab-7 and 4B12

**Fig. 2:**
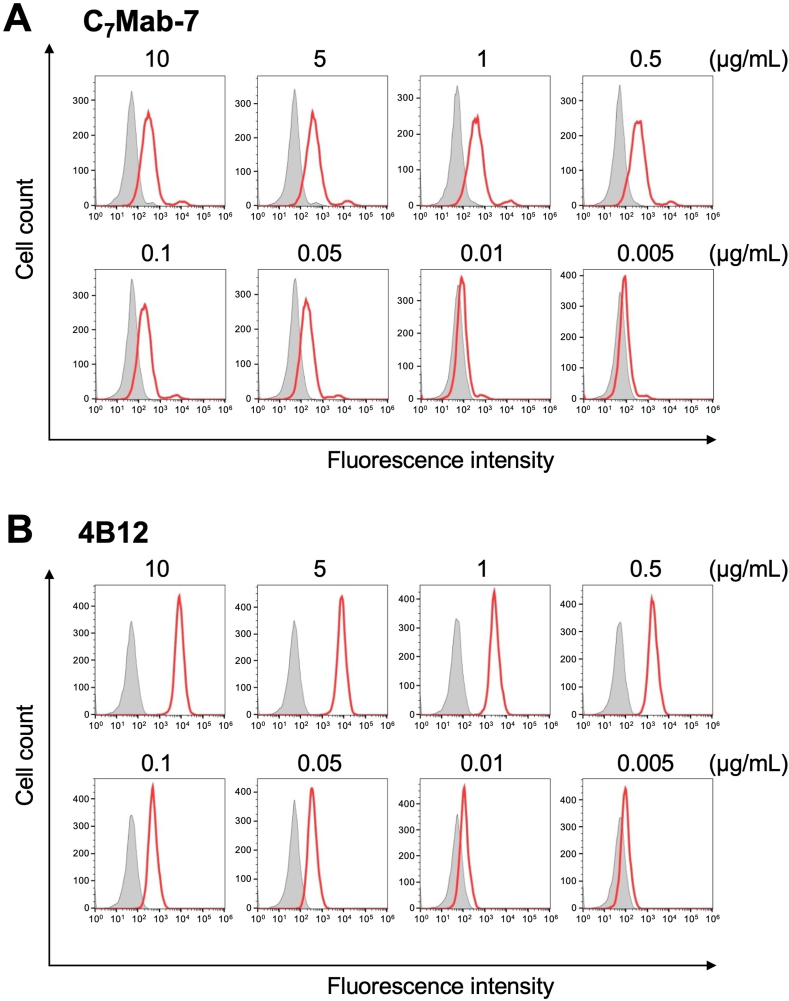
Flow cytometry analysis of anti-mCCR7 mAbs against CHO/mCCR7 and CHO–K1 cells. CHO/mCCR7 (red lines) and CHO–K1 (gray-filled lines) cells were treated with C_7_Mab-7 (A) and a commercially available anti-mCCR7 mAb, 4B12 (B), at the indicated concentrations. Following the treatment, the cells were washed and incubated with anti-rat IgG conjugated with Alexa Fluor 488. Fluorescence data were collected using the SA3800 Cell Analyzer.

The binding of purified C_7_Mab-7 to CHO/mCCR7 and CHO–K1 cells was analyzed using flow cytometry. C_7_Mab-7 exhibited dose-dependent reactivity with CHO/mCCR7 cells at concentrations ranging from 0.005 to 10 μg/mL but did not bind to CHO–K1 cells at any concentration (Fig. 2A). Additionally, a commercially available anti-mCCR7 mAb (4B12) showed higher fluorescence intensity against CHO/mCCR7 cells at concentrations ranging from 0.1 to 10 μg/mL compared to C_7_Mab-7 (Fig. 2B) although the fluorescence intensities of C_7_Mab-7 and 4B12 were comparable at lower concentrations (0.005 to 0.05 μg/mL). These results indicate that C_7_Mab-7 specifically recognized mCCR7 on the cell surface although the fluorescence intensity saturates at mAb concentrations above 0.5 μg/mL in flow cytometry.

### *3.3* *Determination of K*_D_*values of C*_*7*_*Mab-7 and 4B1*2 by *flow cytometry*

**Fig. 3:**
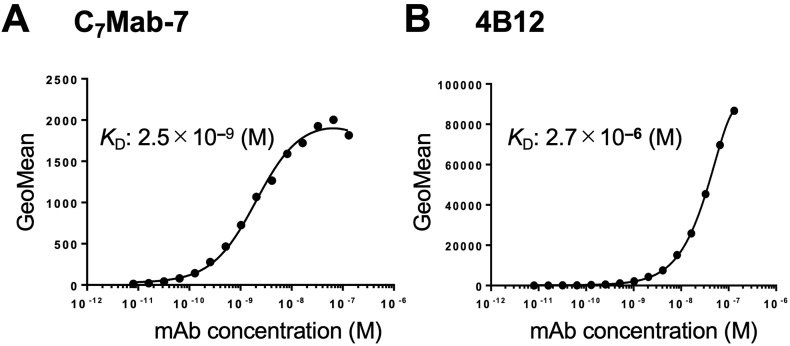
Measurement of the binding affinity of C_7_Mab-7 and 4B12. CHO/mCCR7 cells were treated with serial dilutions of C_7_Mab-7 (A) and 4B12 (B) at the indicated concentrations. Following treatment with the mAbs, the cells were washed and incubated with anti-rat IgG conjugated with Alexa Fluor 488. Fluorescence data were acquired using the SA3800 Cell Analyzer, and the representative graphs were shown. The average *K*_D_ values were determined from two independent measurements.

**Supplementary Fig. S1:**
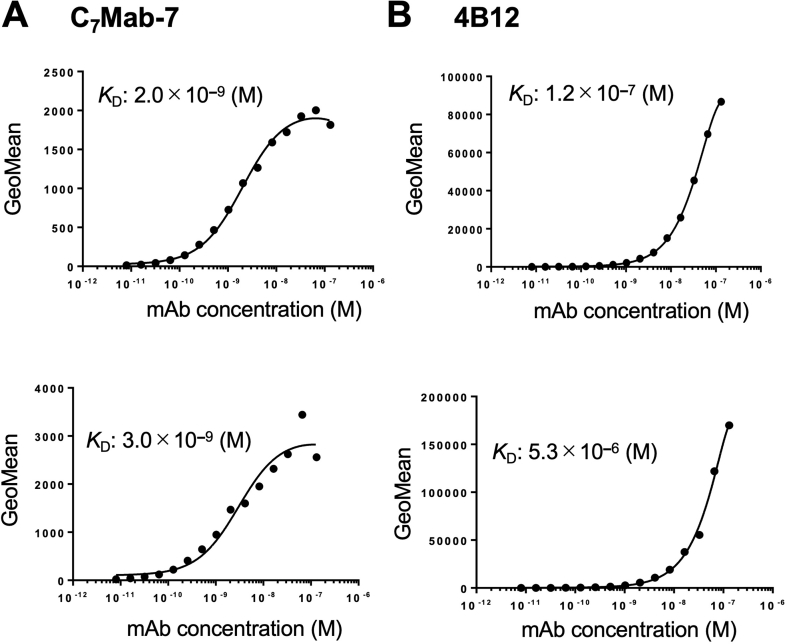
Measurement of the binding affinity of C_7_Mab-7 and 4B12 from two independent measurements. CHO/mCCR7 cells were treated with serial dilutions of C_7_Mab-7 (A) and 4B12 (B) at the indicated concentrations. Following treatment with the mAbs, the cells were washed and incubated with anti-rat IgG conjugated with Alexa Fluor 488. Fluorescence data were acquired using the SA3800 Cell Analyzer. The average *K*_D_ values were determined from two independent measurements in Fig. 3.

The binding affinity of C_7_Mab-7 and 4B12 was evaluated using flow cytometry. The average *K*_D_ value of C_7_Mab-7 for CHO/mCCR7 cells from two independent measurements (Supplementary Fig. S1) was 2.5 × 10⁻⁹ M (Fig. 3), while that of 4B12 for CHO/mCCR7 cells was 2.7 × 10^⁻6^ M. The lower affinity of 4B12 is thought to be due to the intensive fluorescence intensity observed at high concentrations of mAb, which rapidly decreased with lower concentrations of mAb.

### 3.4 Western blot analysis

**Fig. 4:**
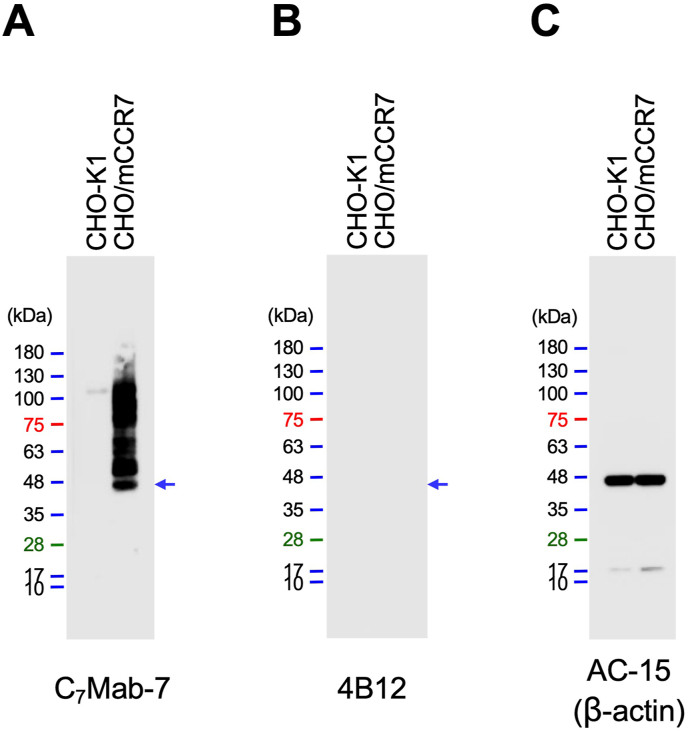
Western blot analysis using C_7_Mab-7. Whole-cell lysates (10 μg/lane) from CHO–K1 and CHO/mCCR7 cells were electrophoresed and transferred onto PVDF membranes. The membranes were incubated with 1 μg/mL of C_7_Mab-7 (A), 4B12 (B), and AC-15 (anti-β-actin mAb) (C). The membranes were subsequently incubated with peroxidase-conjugated anti-rat IgG for C_7_Mab-7 and 4B12 or anti-mouse IgG for AC-15. The blue arrows indicate the estimated molecular weight of mCCR7 (42.9 kDa).

The availability of C_7_Mab-7 for western blot analysis was evaluated using whole cell lysates of CHO–K1 and CHO/mCCR7 cells. C_7_Mab-7 exhibited strong reactivity with mCCR7 at an estimated molecular weight of 42.9 kDa and higher molecular weight positions (Fig. 4A). In contrast, 4B12 did not exhibit any reactivity with mCCR7 (Fig. 4B). These results indicate that C_7_Mab-7 is suitable not only for flow cytometry but also for western blot analysis.

### 3.5 Immunohistochemistry using C_7_Mab-7 in mouse CCR7-overexpressed CHO–K1 cells

**Fig. 5:**
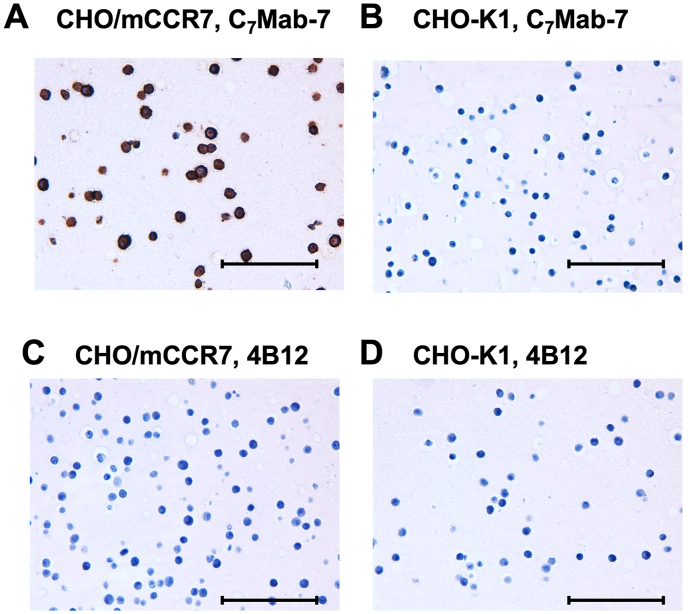
Immunohistochemistry of paraffin-embedded cell sections of CHO/mCCR7 and CHO–K1 cells using C_7_Mab-7 and 4B12 mAbs. Sections of CHO/mCCR7 (A and C) and CHO–K1 (B and D) cells were treated with 1 μg/mL of C_7_Mab-7 (A and B) and 4B12 (C and D), followed by treatment with the Histofine Simple Stain Mouse MAX PO (Rat). Color was developed using DAB, and counterstaining was performed using hematoxylin. Scale bar = 100 μm.

To evaluate the suitability of C_7_Mab-7 for immunohistochemistry in formalin-fixed paraffin-embedded (FFPE) samples, paraffin-embedded sections of CHO/mCCR7 and CHO–K1 cells were stained with C_7_Mab-7. The cytoplasmic and membranous staining of mCCR7 was observed in CHO/mCCR7 cells (Fig. 5A), whereas no staining was detected in CHO–K1 cells (Fig. 5B). No staining was observed in CHO/mCCR7 and CHO-K1 cells treated with 4B12 (Fig. 5C and D).

## 4 Discussion

We developed a mAb against mCCR7 using the CBIS method. The obtained mAb, C_7_Mab-7, is suitable for flow cytometry, western blot, and immunohistochemistry (Fig. 2, Fig. 3, Fig. 4, Fig. 5). The successful establishment of C_7_Mab-7 suggests the presence of an epitope with a stable structure, regardless of the denatured or undenatured state of mCCR7. This is evidenced by the fact that the epitope is recognized in its native conformation in flow cytometry and is detected in a denatured form in western blot. Antibodies that recognize such epitopes have the potential for sensitive detection in immunohistochemistry. Indeed, C_7_Mab-7 is suitable for immunohistochemistry (Fig. 5). Therefore, identifying the epitope of C_7_Mab-7 will facilitate the development of more sensitive anti-CCR7 mAbs, thereby contributing to the advancement of mAb-based therapies and diagnostics.

Western blot analysis of C_7_Mab-7 using CHO/mCCR7 cells showed a band at the estimated molecular weight of 42.9 kDa and bands at higher molecular weight positions (Fig. 4A). A similar band pattern of the western blot was observed in human CCR7-overexpressed HEK293 cells due to the constitutive polyubiquitylation. The ubiquitylation regulates the basal trafficking of CCR7 in the absence of ligands [50]. As shown in Fig. 5A, mCCR7 was detected in both cytoplasm and plasma membrane in immunohistochemistry. Therefore, overexpressed mCCR7 is thought to receive the basal trafficking by ubiquitylation in CHO/mCCR7 cells. C_7_Mab-7 could contribute to the study of the membrane-to-cytoplasm trafficking of mCCR7 through immunofluorescence or antibody-induced receptor internalization studies.

Anti-CCR7 mAbs have already been developed as therapeutic agents for hematologic malignancies [10,35]. Expanding their application to solid cancers requires the efficacy and safety of these mAbs against metastatic cancers in mouse models [51,52]. To target the mCCR7-positive cancer cells using C_7_Mab-7 (rat IgG_1_), generating a class-switched mouse IgG_2a_ mAb from rat IgG_1_ is necessary. We already determined the V_H_ and V_L_ sequences of C_7_Mab-7. Therefore, there is an advantage to produce a large amount of recombinant mAbs for therapeutic uses in preclinical models. Furthermore, generating defucosylated IgG_2a_-type mAbs is also effective for evaluating antibody-dependent cellular cytotoxicity and the *in vivo* antitumor effect in mouse xenograft models [53,54].

In a syngeneic mouse model of oral squamous cell carcinoma, the tumor growth rate was significantly lower in mCCR7-knockout mice compared with the wild-type mice [55]. Single-cell RNA sequence and bioinformatics analyses revealed that the proportion of M2 macrophages in the knockout group was lower than that in the wild-type group [55]. *In vitro* studies showed that mCCR7 can promote M2 macrophage polarization, which promotes the proliferation, invasion, and migration of tumor cells [55]. Therefore, the depletion of mCCR7-positive cells by anti-mCCR7 mAbs like defucosylated IgG_2a_-type C_7_Mab-7 could inhibit tumor growth.

In the unilateral ureteral obstruction model in mice, mCCR7-expressing circulating fibrocytes infiltrate the kidney and contribute to renal fibrosis [56]. The blockade of CCL21/mCCR7 signaling by anti-CCL21 mAbs reduced the renal fibrosis [57]. Therefore, anti-mCCR7 mAbs that block mCCR7 signaling could suppress renal fibrosis. Further studies are required to investigate the neutralizing activity of C_7_Mab-7.

In conclusion, C_7_Mab-7 is a highly sensitive and versatile mAb for basic research and is anticipated to obtain proof-of-concept in preclinical models for the development of antibody therapies.

## CRediT authorship contribution statement

**Hiroyuki Satofuka:** Writing – original draft, Investigation, Funding acquisition. **Hiroyuki Suzuki:** Investigation, Funding acquisition. **Tomohiro Tanaka:** Investigation, Funding acquisition. **Rena Ubukata:** Investigation. **Miu Hirose:** Investigation. **Haruto Yamamoto:** Investigation. **Yu Kaneko:** Investigation. **Shiori Fujisawa:** Investigation. **Guanjie Li:** Investigation. **Mika K. Kaneko:** Funding acquisition, Conceptualization. **Yukinari Kato:** Writing – review & editing, Project administration, Funding acquisition, Conceptualization.

## Author disclosure statement

The authors have no conflicts of interest.

## Funding information

This research was supported in part by 10.13039/100009619Japan Agency for Medical Research and Development (10.13039/100009619AMED) under Grant Numbers: JP24am0521010 (to Y.Kato), JP24ama121008 (to Y.Kato), JP24ama221339 (to Y.Kato), JP23am0401013 (to Y.Kato), JP24ama221339 (to Y.Kato), JP24bm1123027 (to Y.Kato), and JP24ck0106730 (to Y.Kato), and by the 10.13039/501100001691Japan Society for the Promotion of Science (10.13039/501100001691JSPS) Grants-in-Aid for Scientific Research (10.13039/501100001691KAKENHI) grant nos. 24K11652 (to H.Satofuka), 22K06995 (to H.Suzuki), 21K20789 (to T.T.), 21K07168 (to M.K.K.), and 22K07224 (to Y.Kato).

## Declaration of competing interest

The authors declare the following financial interests/personal relationships which may be considered as potential competing interests: Yukinari Kato reports financial support was provided by 10.13039/100009619Japan Agency for Medical Research and Development. Yukinari Kato, Hiroyuki Suzuki reports financial support was provided by 10.13039/501100001691Japan Society for the Promotion of Science. Hiroyuki Satofuka, Tomohiro Tanaka, Mika K. Kaneko, reports financial support was provided by 10.13039/501100001691Japan Society for the Promotion of Science. If there are other authors, they declare that they have no known competing financial interests or personal relationships that could have appeared to influence the work reported in this paper.
